# Modulating Th17/Treg Balance in Alzheimer’s Disease: Therapeutic Insights from Natural Compounds and Immunometabolism

**DOI:** 10.3390/brainsci16050443

**Published:** 2026-04-22

**Authors:** Shuyao Tang, Gangying Fu, Wenjing Yu, Mengfen Zhou, Ting Chen, Zhenyan Song, Shaowu Cheng, Ping Li

**Affiliations:** Key Laboratory of Hunan Province for Integrated Traditional Chinese and Western Medicine on Prevention and Intervention of Cardio-Cerebral Diseases, School of Integrated Chinese and Western Medicine, Hunan University of Chinese Medicine, Changsha 410208, China; 671584396.sy@stu.hnucm.edu.cn (S.T.); 20213763@stu.hnucm.edu.cn (G.F.); wjyu@stu.hnucm.edu.cn (W.Y.); zhouzhou@stu.hnucm.edu.cn (M.Z.); chenting520@hnucm.edu.cn (T.C.); songzhenyan2013@hnucm.edu.cn (Z.S.); scheng@hnucm.edu.cn (S.C.)

**Keywords:** Alzheimer’s disease, Th17/Treg balance, neuroinflammation, immunoregulation, natural compounds

## Abstract

**Highlights:**

**What are the main findings?**

- Th17/Treg imbalance is a central immunological mechanism driving neuroinflammation and cognitive decline in Alzheimer’s disease.
- Natural compounds (e.g., paeoniflorin and ganoderic acid A) can modulate Th17/Treg balance and attenuate AD-related pathology.

**What are the implications of the main findings?**

- Immunometabolic regulation provides a unifying framework linking metabolism to Th17/Treg imbalance in AD.
- Most current evidence is derived from preclinical or non-AD models, highlighting limitations in direct translational relevance.
- Targeting Th17/Treg balance represents a promising immunomodulatory strategy, but clinical validation remains limited.

**Abstract:**

**Background/Objectives:** Alzheimer’s disease (AD) is a neurodegenerative disorder characterized by progressive cognitive decline and chronic neuroinflammation. Increasing evidence suggests that the imbalance between pro-inflammatory Th17 cells and anti-inflammatory regulatory T (Treg) cells plays a critical role in AD pathogenesis. However, a comprehensive synthesis of how natural compounds modulate Th17/Treg balance in AD remains lacking. This review aims to summarize current preclinical evidence on Th17/Treg dysregulation and evaluate the immunomodulatory potential of natural compounds in AD. **Methods:** This review focuses on preclinical evidence derived from experimental AD models and related inflammatory models to evaluate how natural compounds modulate Th17/Treg balance, neuroinflammation, and cognitive function, with an emphasis on underlying molecular and immunometabolic mechanisms. **Results:** Th17/Treg imbalance contributes significantly to AD-associated neuroinflammation and disease progression. Representative natural compounds, including paeoniflorin, quercetin, and ganoderic acid A, have demonstrated the ability to rebalance Th17/Treg responses, suppress neuroinflammation, and improve neuronal survival in experimental models. These compounds are highlighted due to their relatively stronger evidence in AD-related models and more clearly defined immunomodulatory mechanisms. These effects are partially mediated through modulation of key signaling pathways and immunometabolic reprogramming. **Conclusions:** Targeting Th17/Treg balance with natural compounds represents a promising multi-target immunomodulatory strategy for AD. However, most current evidence is derived from preclinical or non-AD models, and clinical validation remains limited. Future studies should prioritize AD-specific models and translational research to evaluate therapeutic potential in humans.

## 1. Introduction

Alzheimer’s disease (AD) is a multifactorial neurodegenerative disorder and one of the leading causes of disability in the aging population worldwide. Its increasing prevalence poses a major threat to public health and imposes substantial socioeconomic burdens. Notably, AD and related dementias are currently ranked as the seventh leading cause of death globally [1]. Although the etiopathogenesis of AD remains incompletely understood, accumulating evidence highlights neuroinflammation as a central driver of disease progression. Recent studies have emphasized the critical involvement of the immune system, particularly peripheral immune responses, in AD pathogenesis. Among these, the imbalance of T-cell subsets—especially the disrupted equilibrium between pro-inflammatory Th17 cells and anti-inflammatory regulatory T (Treg) cells—has emerged as a key mechanism underlying sustained neuroinflammation [2]. The blood–brain barrier (BBB) dysfunction facilitates the infiltration of peripheral immune cells into the central nervous system (CNS), further amplifying inflammatory responses [3]. Within this context, Th17/Treg imbalance promotes excessive cytokine production, microglial activation, and progressive neuronal damage. Despite increasing recognition of adaptive immune dysregulation in AD, a comprehensive and critical synthesis of how natural compounds modulate Th17/Treg balance remains lacking. Moreover, the emerging role of immunometabolic reprogramming in shaping Th17/Treg differentiation has not been systematically integrated into current AD research frameworks [4].

**Figure A1:**
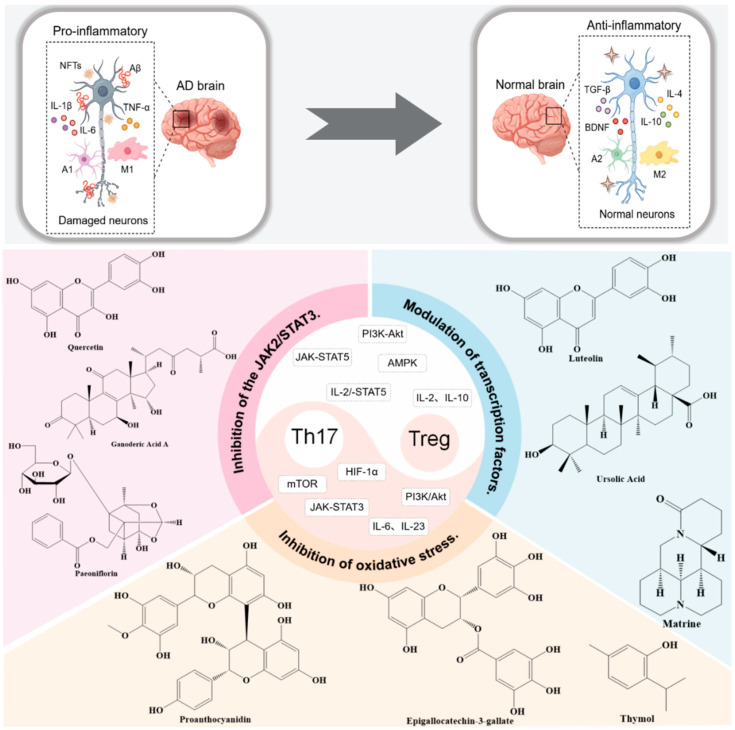
Natural compounds regulate neuroinflammation and Th17/Treg balance in AD.

Therefore, this review focuses on preclinical evidence derived from experimental AD models and related inflammatory models to evaluate how natural compounds regulate Th17/Treg balance, neuroinflammation, and cognitive function. By integrating molecular signaling and immunometabolic mechanisms, this review aims to provide a conceptual framework for understanding immune-targeted therapeutic strategies and to highlight key limitations and future directions for clinical translation in AD.An overview of the regulatory effects of natural compounds on neuroinflammation and Th17/Treg balance in AD is illustrated in Figure A1 (see in Appendix A).

## 2. Pathological Basis and Neuroinflammatory Mechanisms in AD

### 2.1. Overview of Alzheimer’s Disease

With the rapid expansion of the aging population, neurodegenerative disorders, particularly AD, represent a major public health challenge. Epidemiological data indicate that patients with AD in China account for nearly 25% of the global dementia population, making it the country with the highest disease burden [5]. Globally, the prevalence of AD is projected to reach approximately 130 million cases by 2050 [6]. The pathological hallmarks of AD are the extracellular deposition of amyloid-β (Aβ) peptides and the intraneuronal accumulation of neurofibrillary tangles (NFTs).

Currently available pharmacological interventions for AD can be broadly categorized into 3 classes: cholinergic agents, N-methyl-D-aspartate (NMDA) receptor antagonists, and non-competitive Aβ antibodies [7]. Widely used drugs include cholinesterase inhibitors (donepezil, galantamine, and rivastigmine) and the NMDA receptor antagonist memantine. These agents provide partial symptomatic relief by improving cognitive performance and preserving activities of daily living [8]. However, none of the existing treatments can halt or reverse disease progression. The pathogenesis of AD is multifactorial and involves interrelated mechanisms, including extracellular Aβ deposition, intraneuronal NFT accumulation, oxidative stress, neuroinflammation, synaptic dysfunction, and neuronal apoptosis [9]. In addition, dysregulated cytokine networks and aberrant activation of downstream signaling pathways are critically implicated in both the initiation and progression of AD pathology [10].

### 2.2. Neuroinflammation in AD

Neuroinflammation is now recognized as a central contributor to AD pathogenesis, alongside Aβ deposition and neurofibrillary tangles. Accumulating evidence indicates that Aβ aggregation triggers sustained inflammatory responses and oxidative stress, thereby amplifying neuronal dysfunction and disease progression. Neuroinflammation in AD is characterized by persistent activation of the innate immune system, progressive neurodegeneration, and pronounced glial reactivity.

Astrocytes and microglia, the principal immune effector cells in the CNS, play key roles in shaping the inflammatory microenvironment [11]. Postmortem studies of AD brains reveal dense clusters of reactive microglia and astrocytes surrounding Aβ plaques, highlighting their pivotal role in the inflammatory microenvironment [12]. In early stages, these cells contribute to tissue homeostasis and debris clearance; however, with disease progression, they undergo maladaptive activation, leading to excessive production of pro-inflammatory mediators [13]. This dysregulated response interacts with oxidative stress, forming a self-amplifying cycle that drives chronic neuroinflammation and neuronal injury [14].

### 2.3. The Role of Glial Cells in Neuroinflammation

#### 2.3.1. Microglia

Microglia are the resident immune cells of the CNS and serve as primary regulators of neuroinflammation in AD [15]. Under physiological conditions, microglia maintain homeostasis through immune surveillance and phagocytosis. In AD, however, persistent stimulation by Aβ aggregates drives chronic activation, shifting microglial functions from neuroprotection to neurotoxicity [16]. Activated microglia exhibit phenotypic heterogeneity, broadly categorized into pro-inflammatory (M1) [17] and anti-inflammatory (M2) phenotypes [18]. M1 microglia promote neuroinflammation by releasing cytokines such as interleukin-1β (IL-1β), interleukin-6 (IL-6), and tumor necrosis factor-α (TNF-α), whereas M2 phenotypes contribute to anti-inflammatory responses and tissue repair by releasing interleukin-10 (IL-10) and interleukin-4 (IL-4) [19]. This functional dichotomy underscores the dual role of microglia in AD pathogenesis [20]. This dynamic polarization critically determines the balance between neuroprotection and neurotoxicity.

Microglial activation is mediated by several receptors, including Toll-like receptors (TLRs), scavenger receptors, complement receptors, the receptor for advanced glycation end products (RAGE), and triggering receptors expressed on myeloid cells 2 (TREM2) [21]. Among these, TREM2 plays a key role in regulating microglial survival, migration, and phagocytosis. Impaired TREM2 signaling disrupts Aβ clearance and promotes pro-inflammatory activation, thereby exacerbating neurodegeneration [22]. Conversely, TREM2 upregulation promotes M2 polarization and neuroprotection, while its loss skews microglia toward a harmful M1 phenotype [23]. Sustained microglial activation establishes a pathogenic feedback loop, in which Aβ accumulation further amplifies inflammatory signaling, oxidative stress, and neuronal damage [24,25]. While microglia initially exert protective effects, their chronic dysregulation drives persistent neuroinflammation and cognitive decline, highlighting microglial polarization as a potential therapeutic target [26].

#### 2.3.2. Astrocyte

Astrocytes are essential for maintaining neuronal homeostasis, regulating synaptic activity, and preserving BBB integrity [27]. Astrocytes remove Aβ through receptors such as low-density lipoprotein receptor-related protein 1 (LRP1) and scavenger receptor class B member 1 (SCARB1) [28], followed by degradation via neprilysin (NEP), insulin-degrading enzymes (IDEs), and matrix metalloproteinase-9 (MMP9) through the endosomal-lysosomal pathway [29]. Astrocyte-derived apolipoprotein E (APOE) further promotes parenchymal Aβ clearance through LRP1-dependent transport [30].

**Figure A2:**
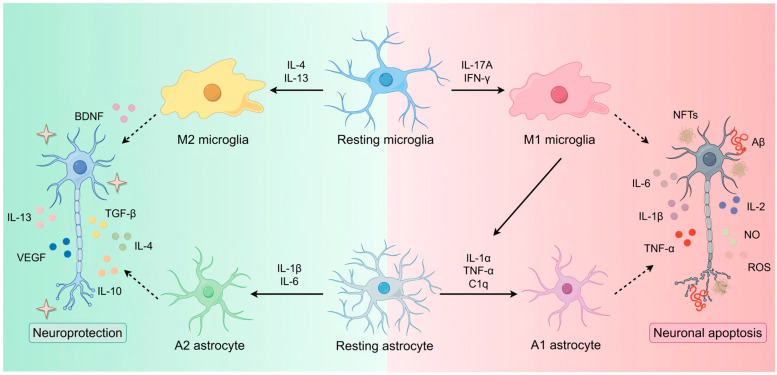
Activation Phenotypes and Secretory Profiles of Microglia and Astrocytes.

**Figure A3:**
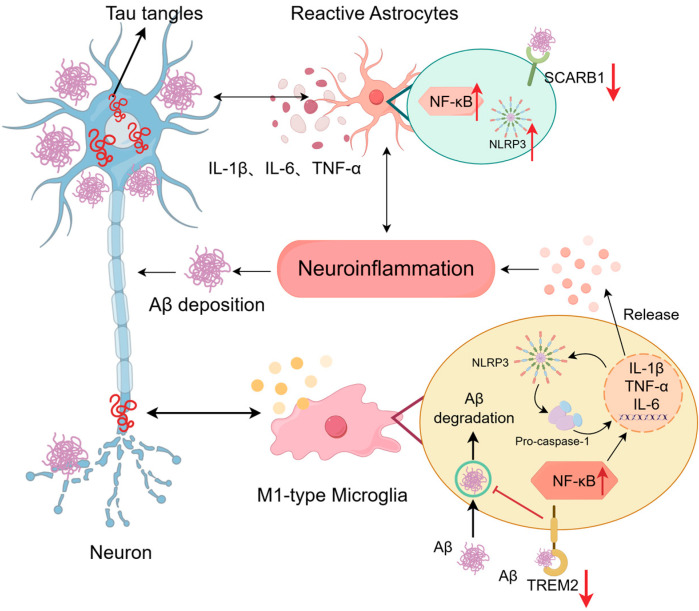
Aβ/Tau-Induced Glial Activation and Inflammatory Cascade Drive AD Progression.

In AD, astrocytes undergo reactive transformation in response to microglia-derived cytokines and Aβ deposition [31,32]. Activated astrocytes enhance NF-κB signaling and produce pro-inflammatory mediators, including IL-1β, IL-6, and TNF-α, thereby amplifying neuroinflammation [33]. In addition, astrocytic dysfunction also disrupts BBB integrity. A compromised BBB function enhances β- and γ-secretase activity, increasing Aβ production, and impaired clearance [34]. These alterations further promote oxidative stress and tau pathology, forming a synergistic network that accelerates neurodegeneration [35]. Thus, astrocytes function as critical regulators of neuronal homeostasis under physiological conditions, but their dysfunction in AD leads to impaired Aβ clearance, persistent neuroinflammation, and BBB breakdown. These interrelated changes act synergistically to exacerbate the progression of AD pathology.The activation phenotypes of glial cells and the Aβ/tau-induced inflammatory cascade in AD are illustrated in Figure A2 and Figure A3 (see in Appendix A).

## 3. Th17/Treg Balance and Factors Influencing Th17/Treg Balance

T helper 17 (Th17) cells and regulatory T (Treg) cells share a reciprocal developmental pathway but exhibit functional antagonism that maintains immune homeostasis [36]. Naïve CD4^+^ T cells differentiate into either Th17 or Treg subsets depending on the cytokine milieu, thereby exerting divergent immunological roles. Th17 cells arise in the presence of interleukin IL-6, IL-21, IL-22, IL-23, and TGF-β [37], with differentiation governed by the transcription factors retinoic acid-related orphan receptor gamma t (RORγt) and signal transducer and activator of transcription 3 (STAT3) [38]. Functionally, Th17 cells produce the signature cytokine IL-17 and display robust pro-inflammatory activity, contributing to tissue inflammation and autoimmune pathology [39].

Conversely, Treg cells differentiate under the influence of TGF-β and IL-2, characterized by expression of the lineage-defining transcription factor forkhead box protein 3 (Foxp3) [40]. They exert immunosuppressive effects primarily through the secretion of anti-inflammatory cytokines such as IL-10 and IL-35, thereby restraining excessive immune activation and preserving tolerance. Emerging evidence further suggests that AD-related pathological factors, including Aβ accumulation and tau pathology, can disrupt Th17/Treg balance by promoting pro-inflammatory signaling and impairing regulatory immune responses [41]. In addition, oxidative stress, a key feature of AD, has been shown to favor Th17 differentiation while destabilizing Treg function, thereby exacerbating immune dysregulation [42].

### 3.1. Cytokines

Cytokines play a central role in regulating Th17/Treg differentiation by shaping the local immune microenvironment. Naïve CD4^+^ T cells differentiate into either pro-inflammatory Th17 cells or anti-inflammatory Treg cells depending on cytokine signals [43]. Evidence indicates that the Th17/Treg balance is critically regulated by inflammatory cytokines, with key mediators including TGF-β, IL-2, IL-6, IL-15, IL-18, IL-21, and IL-23 [44].

TGF-β acts as a key bifunctional regulator of both lineages. In the absence of pro-inflammatory cytokines, TGF-β promotes Treg differentiation through Smad2/3-mediated upregulation of Foxp3 [45]. However, in the presence of inflammatory cytokines such as IL-6 and TGF-β, they instead drive Th17 differentiation by inducing RORγt expression [46].

IL-2 is essential for Treg development and function through activation of STAT5 and upregulation of Foxp3 [47]. At the same time, IL-2 suppresses Th17 differentiation by inhibiting IL-6 receptor signaling, thereby maintaining immune balance [48].

IL-6, together with IL-21 and IL-23, plays a critical role in promoting Th17 lineage commitment. IL-6 represents a critical initiator of Th17 differentiation from naïve CD4^+^ T cells. Its presence drives Th17 commitment, whereas its absence promotes Treg development [49]. IL-21 [50] and IL-23 [51] activate STAT3 signaling, which induces RORγt expression and enhances transcription of Th17-associated genes, including IL-17 and IL-23R, thereby reinforcing pro-inflammatory responses.

In addition to the core cytokines, several auxiliary factors also contribute to the fine regulation of Th17/Treg balance. For example, IL-18 has been reported to inhibit Th17 differentiation by modulating MyD88-dependent IL-1R signaling [52]. IL-2 [53], together with IL-15, promotes Treg differentiation via STAT5-mediated upregulation of Foxp3 and can suppress Th17 responses by reducing IL-17 production [54].

Beyond cytokines, metabolic regulators such as hypoxia-inducible factor 1α (HIF-1α) further modulate Th17/Treg balance [49]. As a key effector of mTOR signaling, HIF-1α drives metabolic reprogramming toward glycolysis, supporting Th17 development [55]. HIF-1α enhances Th17 commitment through transcriptional activation of RORC and concurrently destabilizes Foxp3 via direct binding, thereby suppressing Treg differentiation [56].

Collectively, cytokine-mediated signaling networks tightly regulate Th17/Treg differentiation. Under inflammatory conditions, such as those observed in AD, this regulatory balance is often disrupted, leading to a Th17-dominant immune response that contributes to sustained neuroinflammation.

### 3.2. Signaling Pathway

#### 3.2.1. mTOR Signaling Pathway

The mechanistic target of rapamycin (mTOR), a phosphoinositide 3-kinase (PI3K)-related kinase, functions as a central metabolic sensor that coordinates cellular energy responses and modulates helper T cell development and effector functions [57]. mTOR forms two distinct complexes: mTOR complex 1 (mTORC1) and mTOR complex 2 (mTORC2). mTORC1 activity promotes Th1 and Th17 differentiation, whereas mTORC2 supports Th2 cell development. Both complexes negatively regulate Treg generation through distinct mechanisms [58]. Overall, mTOR signaling skews T cell differentiation toward pro-inflammatory phenotypes by enhancing Th17 expansion and limiting Foxp3-mediated Treg development, highlighting its central role in Th17/Treg imbalance [59].

#### 3.2.2. PI3K/Akt Signaling Pathway

The PI3K/Akt signaling pathway acts upstream of mTOR. Following T cell receptor activation, PI3K phosphorylates and catalyzes the production of phosphatidylinositol-3,4,5-trisphosphate (PIP3). Akt is subsequently phosphorylated and activated through interactions with PIP3 and 3-phosphoinositide-dependent protein kinase-1 (PDK1), which mediates downstream targets including FoxO1 [60]. In Treg cells, FoxO1 is essential for maintaining Foxp3 expression, whereas PI3K/Akt activation promotes glycolytic reprogramming and favors Th17 differentiation [61]. Inhibition of PI3K/Akt signaling suppresses Th17 development and promotes Treg generation [62]. Thus, this pathway contributes to Th17/Treg imbalance primarily through metabolic reprogramming and amplification of inflammatory responses [63].

#### 3.2.3. AMPK Signaling Pathway

AMP-activated protein kinase (AMPK) functions as a metabolic checkpoint that counteracts mTOR signaling. By inhibiting mTORC1 activity, AMPK suppresses glycolysis-dependent Th17 differentiation while promoting Treg generation [64]. In Treg cells, AMPK enhances fatty acid oxidation and mitochondrial metabolism, thereby supporting their proliferation and suppressive function [65]. Pharmacological activation of AMPK, such as with metformin, has been shown to inhibit Th17 differentiation and promote Treg expansion [66]. In contrast to mTOR signaling, AMPK exerts a protective role in maintaining immune homeostasis by shifting the balance toward anti-inflammatory Treg responses.

#### 3.2.4. JAK/STAT Signaling Pathway

The Janus kinase (JAK)/signal transducer and activator of transcription (STAT) pathway is a central regulator of cytokine-mediated T cell differentiation [67]. Upon phosphorylation, STAT3 forms dimers that translocate to the nucleus and bind regulatory regions of the RORγt gene, thereby promoting Th17 differentiation and enhancing transcription of Th17-associated genes [68,69]. STAT5 activation downstream of IL-2 signaling drives Foxp3 expression and promotes Treg development [70,71]. During inflammatory conditions, excessive IL-6/STAT3 signaling suppresses Foxp3 and skews differentiation toward Th17 cells, resulting in enhanced pro-inflammatory responses [72]. Among the signaling pathways discussed, the STAT3–STAT5 axis represents one of the most direct and well-characterized mechanisms governing Th17/Treg lineage commitment.

Collectively, these signaling pathways form an interconnected regulatory network in which metabolic cues and cytokine signaling converge to determine Th17/Treg balance. Notably, mTOR and PI3K/Akt pathways predominantly promote Th17 differentiation, whereas AMPK and STAT5 signaling favor Treg development. This coordinated regulation provides a mechanistic basis for targeting Th17/Treg imbalance in AD and other inflammatory conditions.A detailed summary of cytokines involved in Th17/Treg regulation is provided in Table A1 (see in Appendix A).

### 3.3. Metabolic Regulation

T cell metabolism is highly dynamic and plays a central role in regulating immune cell function. Distinct metabolic programs underlie the differentiation and functional specialization of Th17 and Treg cells, forming the basis of immunometabolic regulation [73].

Th17 cell development and effector functions are highly dependent on glycolysis, glutaminolysis, and de novo fatty acid synthesis. Enhanced glycolytic flux supports rapid energy production and biosynthesis [74], accompanied by upregulation of glucose metabolism-related genes and accumulation of glycolytic intermediates such as pyruvate, lactate, and pentose phosphate pathway products, thereby promoting Th17 differentiation [75]. In addition, increased expression of acetyl-CoA carboxylase 1 (ACC1) enhances RORγt-mediated transcriptional activation at the IL-17 locus, further promoting Th17 lineage commitment [76]. Th17 cells also exhibit elevated glutaminolytic activity, leading to increased production of α-ketoglutarate (α-KG), which supports RORγt-driven transcription [77,78]. This metabolic shift is partly mediated by upregulation of inducible cAMP early repressor (ICER), which enhances glutaminase 1 expression and glutamine catabolism [79]. Moreover, glutamine-derived metabolites such as 2-hydroxyglutarate contribute to epigenetic regulation by repressing Foxp3 expression through promoter hypermethylation, thereby reinforcing Th17 differentiation and disrupting Th17/Treg balance [80]. In addition, oxidative stress further promotes Th17 differentiation while impairing Treg stability, thereby amplifying Th17/Treg imbalance under inflammatory conditions.

In contrast, Treg cells primarily rely on oxidative phosphorylation and fatty acid oxidation (FAO) to sustain their energy demands [81]. FAO, a mitochondrial process dependent on carnitine palmitoyltransferase 1 (CPT1), is highly active in Tregs and supports mitochondrial function and Foxp3 stability, thereby promoting Treg proliferation and immunosuppressive activity [82]. In addition, the mevalonate pathway plays a critical role in Treg maintenance, as its disruption markedly reduces Treg abundance [83]. Activation of this pathway enhances Smad3 phosphorylation and potentiates TGF-β signaling, thereby promoting Foxp3 expression and sustaining Treg function [84]. These metabolic programs are essential for maintaining Treg stability and suppressive capacity under inflammatory conditions. Disruption of FAO or mitochondrial metabolism impairs Treg function and shifts immune balance toward pro-inflammatory responses.

Importantly, metabolic pathways act as key regulatory checkpoints linking environmental cues to immune cell fate. Interventions that inhibit glycolysis or activate FAO have been shown to suppress Th17 differentiation while promoting Treg development. Collectively, these findings indicate that metabolic reprogramming is a critical determinant of Th17/Treg balance. In the context of AD, where metabolic dysregulation and oxidative stress are prominent, targeting immunometabolic pathways may provide a promising strategy for restoring immune homeostasis.

**Figure A4:**
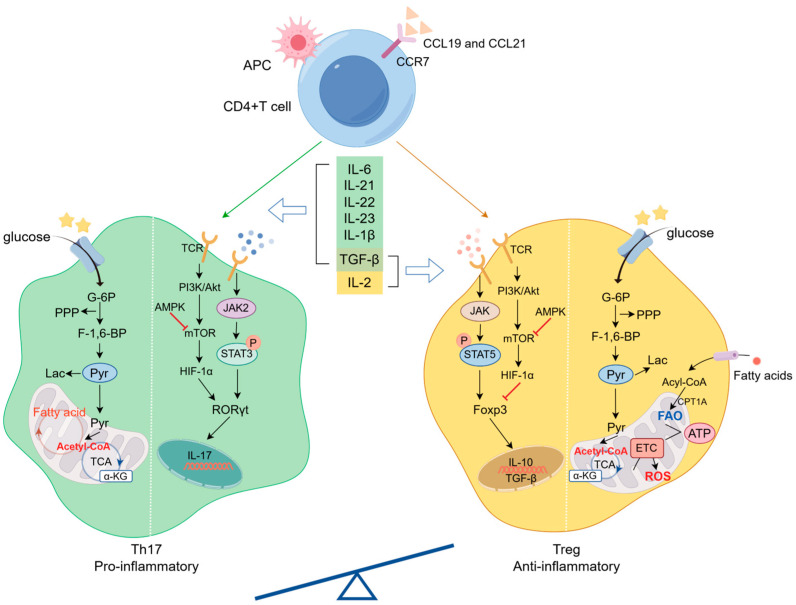
Multidimensional Regulation of Th17/Treg Balance.

However, most current evidence is derived from preclinical studies, and the translational relevance of immunometabolic regulation in AD remains to be fully established.The multidimensional regulatory mechanisms underlying Th17/Treg balance are summarized in Figure A4 (see in Appendix A).

## 4. The Critical Role of Th17/Treg Balance in AD

### 4.1. CD4^+^ T Lymphocytes and AD

Beyond glial cells, adaptive immune components—particularly CD4^+^ T lymphocytes—play an important role in AD-associated neuroinflammation. Under physiological conditions, the BBB restricts peripheral CD4^+^ T cell entry into the CNS. However, in AD, BBB dysfunction facilitates the infiltration of activated CD4^+^ T cells into the brain parenchyma, where they accumulate in regions characterized by severe neurodegeneration and tau pathology [85]. Neuropathological studies have demonstrated increased densities of CD4^+^ T cells in AD brains compared with non-AD dementia and healthy controls, accompanied by alterations in their activation status and subset composition [86]. This suggests that BBB dysfunction occurs in AD brains, allowing CD4^+^ T cells to migrate into the brain parenchyma and mediate neuroinflammation. Aβ accumulation and neuronal injury contribute to this process by inducing cerebrovascular endothelial damage and triggering the release of chemotactic signals. For example, chemokines such as CCL19 and CCL21 can activate CCR7-expressing CD4^+^ T cells, promoting their recruitment and activation [87]. In addition, Aβ can enter the peripheral circulation and be recognized by antigen-presenting cells (APCs), thereby activating systemic immune responses and driving CD4^+^ T cell differentiation [88]. Once activated, infiltrating CD4^+^ T cells further amplify neuroinflammation through the release of pro-inflammatory cytokines [2]. This bidirectional interaction provides a mechanistic link between systemic immune dysregulation and Th17/Treg imbalance in AD.

### 4.2. Interactions Between Th17/Treg Cells and Neuroglial Cells

Under inflammatory conditions, CD4^+^ T cells preferentially differentiate into Th17 cells, whereas under homeostatic conditions, they give rise to Treg cells that suppress excessive immune activation [89]. In AD, this balance is disrupted, leading to Th17 polarization and reduced Treg-mediated immunoregulation [90]. Accumulating evidence indicates that immune mechanisms play a fundamental role in cognitive impairment, supporting the view that AD involves systemic immune dysregulation [91]. Clinical studies and animal models consistently demonstrate increased RORγt expression and reduced Foxp3 levels [92], indicating a pronounced Th17/Treg imbalance associated with cognitive decline and disease severity [93].

Upon infiltration into the CNS, Th17 cells promote neuroinflammation through multiple coordinated mechanisms. IL-17 activates microglia via IL-17/IL-17R signaling, upregulating immunoregulatory molecules such as Intercellular adhesion molecule 1 (ICAM-1), Cluster of differentiation 40 (CD40), Cluster of differentiation 80 (CD80), and Cluster of differentiation 86 (CD86), as well as Major histocompatibility complex class II (MHC-II), and inducing the release of pro-inflammatory mediators including IL-1β, TNF-α, IL-6, complement components, and ROS [94]. These cytokines contribute to neuronal apoptosis and progressive neurodegeneration [95]. In parallel, astrocytes respond to IL-17 by adopting a reactive phenotype and producing inflammatory cytokines and chemokines, further amplifying neuroinflammatory responses [96]. Th17-associated cytokines can also upregulate adhesion molecules such as vascular cell adhesion molecule-1 (VCAM-1), facilitating immune cell recruitment into the CNS [97].

Importantly, these processes form a self-amplifying inflammatory loop [98]. Aβ and tau pathology stimulate microglia to produce ROS and nitric oxide, enhancing oxidative stress and further promoting Th17 differentiation and IL-17 production [99]. Meanwhile, IL-17 disrupts BBB integrity, allowing the entry of additional peripheral immune cells and neurotoxic mediators, thereby exacerbating neurodegeneration [100]. Elevated levels of Th17-associated cytokines, including IL-17A, IL-21, and IL-23, have been detected in both AD models [101,102] and patients [103], suggesting coordinated pro-inflammatory signaling that contributes to disease progression. This systemic inflammatory amplification further exacerbates neuroinflammation and neuronal damage. Consequently, targeting Th17-driven responses to Aβ may represent a promising strategy to attenuate neuroinflammatory cascades and slow disease progression [104].

In contrast, Treg cells exert immunomodulatory and neuroprotective effects by secreting anti-inflammatory cytokines such as IL-10, IL-35, and TGF-β. These cytokines suppress microglial activation and maintain immune homeostasis [105]. Preclinical studies demonstrate that Treg expansion or adoptive transfer reduces amyloid plaque burden, attenuates neuroinflammation, and improves cognitive function, whereas Treg depletion exacerbates disease progression [106]. Treg cells can also promote the polarization of microglia toward an anti-inflammatory phenotype, representing a key mechanism for limiting neuroinflammation [107]. However, the protective effects of Treg cells appear to be stage-dependent. In early AD, BBB disruption may facilitate Treg infiltration and transient immune protection [108]. In later stages, sustained inflammatory signaling, particularly IL-17 production, impairs Treg function [109], leading to diminished immunosuppressive capacity [110]. Clinical studies further report both quantitative and functional deficits in circulating Treg cells, highlighting a progressive loss of immune regulation during AD progression [2].

Consequently, Treg reconstitution has emerged as a promising therapeutic strategy for neurodegenerative diseases, with ongoing research focused on clinical approaches for Treg expansion [111]. Aβ and tau pathology not only drive neuroinflammation but also actively promote Th17 polarization and impair Treg function, thereby contributing to Th17/Treg imbalance [112]. Experimental evidence indicates that Treg ablation worsens cognitive function in mice, whereas low-dose IL-2 therapy to enhance Treg populations shows potential to delay neurodegeneration, possibly through microglia-mediated Aβ clearance [113]. Supporting this concept, adoptive transfer of Treg cells in AD models has been shown to improve cognitive function, reduce amyloid deposition, and attenuate neuroinflammatory responses, underscoring the critical role of Treg-mediated immunomodulation in AD pathophysiology [114].

**Figure A5:**
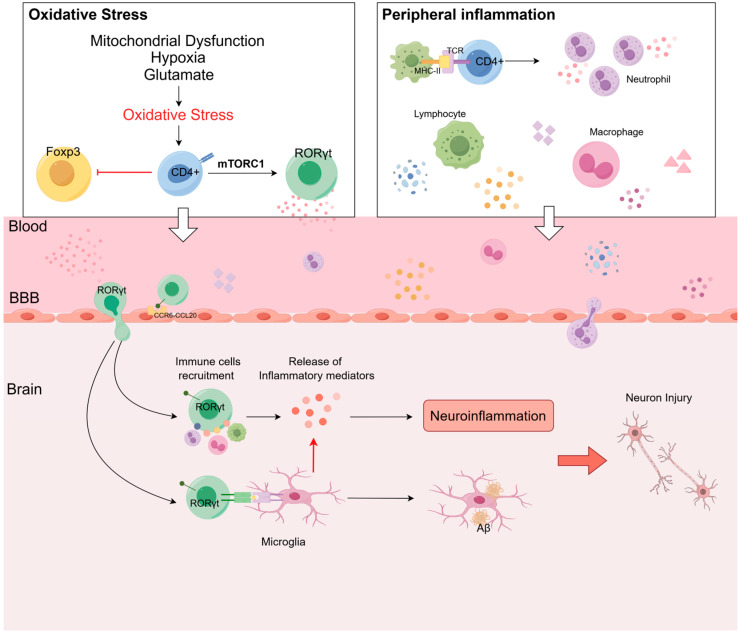
Mechanisms of Th17/Treg imbalance-induced neuroinflammation in AD.

In addition, evidence from genetically modified models suggests a potential role of Th17/Treg imbalance in neuroinflammatory processes, although its specific contribution to AD requires further investigation. Overall, Th17/Treg imbalance represents a central immunopathological mechanism in AD. The interplay between Th17-driven inflammation, Treg dysfunction, and neuroglial activation establishes a pathogenic feedback loop linking peripheral immune dysregulation to chronic neuroinflammation and neurodegeneration [115].The mechanisms by which Th17/Treg imbalance drives neuroinflammation in AD are illustrated in Figure A5 (see in Appendix A).

## 5. Research Advances in Natural Compounds Targeting Th17/Treg Balance-Mediated Neuroinflammation in AD

### 5.1. Overview and Advantages of Natural Compounds

Natural medicines consist of pharmacologically active compounds derived from animal, plant, and mineral sources. These substances have historically played a critical role in drug discovery. Although no current therapies effectively slow the progression of AD [116], natural compounds are increasingly recognized as promising candidates for anti-AD strategies due to their favorable safety profiles and low toxicity. As the seventh leading cause of death worldwide, AD involves multifaceted pathological mechanisms. Natural medicines offer distinct advantages for multi-target intervention, capable of simultaneously modulating oxidative stress, neuroinflammatory responses, Aβ aggregation, and tau hyperphosphorylation [117].

In contrast, conventional synthetic drugs, including cholinesterase inhibitors (e.g., donepezil) and NMDA receptor antagonists (e.g., memantine), typically target single pathological pathways. Their limited scope often results in insufficient efficacy and undesirable side effects, such as gastrointestinal disturbances, dizziness, and hallucinations [118,119]. Natural medicines, on the other hand, are associated with improved safety, reduced production costs, and more economical long-term treatment options.

### 5.2. Natural Medicines Targeting AD Neuroinflammation via Th17/Treg Balance

A growing body of evidence indicates that natural compounds can modulate Th17/Treg balance and attenuate neuroinflammation through multiple mechanisms, including regulation of key signaling pathways, cytokine networks, and oxidative stress. Rather than acting on a single target, these compounds typically exert multi-layered immunomodulatory effects, highlighting their potential as therapeutic candidates for AD.

#### 5.2.1. Targeting the JAK/STAT Signaling Pathway

The JAK/STAT pathway plays a central role in regulating Th17/Treg differentiation, particularly through STAT3-mediated Th17 activation. Several natural compounds have been reported to modulate this pathway and restore immune balance.

Ganoderic acid A (GAA), a lanostane-type triterpenoid isolated from Ganoderma lucidum, has shown therapeutic efficacy in AD models by restoring Th17/Treg homeostasis and reducing neuroinflammatory responses. Mechanistically, GAA suppresses Th17 activation through inhibition of JAK2/STAT3 signaling while enhancing Treg function, ultimately contributing to improved cognitive performance in D-galactose-induced aging mice [120].

Similarly, paeoniflorin (PF) exerts multi-target immunomodulatory effects. PF inhibits STAT3 phosphorylation in hippocampal neurons, leading to reduced expression of Th17-associated cytokines such as IL-17A, IL-17F, and IL-22, as well as TNF-α [121]. In addition, PF regulates apoptosis-related proteins by modulating Bcl-2 and Bax expression, thereby protecting against glutamate-induced neurotoxicity [122]. Beyond JAK/STAT signaling, PF also suppresses Th17 differentiation via inhibition of NF-κB and ERK pathways, reducing TNF-α-dependent chemokine production and leukocyte migration [123].

Quercetin also interferes with JAK/STAT signaling by inhibiting IL-12-induced tyrosine phosphorylation of JAK2 and TYK2, thereby reducing downstream STAT3/STAT4 activation. In addition to its immunomodulatory effects, quercetin exhibits potent antioxidant activity, attenuates oxidative stress, inhibits Aβ aggregation, and promotes Aβ clearance, collectively mitigating Aβ-induced neurotoxicity and neuroinflammation [124,125,126]. However, it should be noted that most evidence for quercetin and paeoniflorin is derived from non-AD models, which may limit its direct translational relevance.

#### 5.2.2. Targeting Th17/Treg-Related Cytokine Signaling and Transcriptional Regulation

Compounds modulating Th17/Treg-associated cytokines and transcriptional programs further contribute to immune rebalancing in AD.

Matrine, a quinolizidine alkaloid, has demonstrated efficacy in AD models by restoring Th17/Treg homeostasis and improving cognitive function. Mechanistically, matrine suppresses pro-inflammatory cytokines such as IL-17A and IL-23 while enhancing anti-inflammatory mediators, including TGF-β and IL-35. It also regulates lineage-specific transcription factors by downregulating RORγt and upregulating Foxp3, thereby inhibiting Th17 differentiation and promoting Treg development. In addition, matrine reduces TNF-α and IL-1β levels and suppresses microglial activation, further attenuating neuroinflammation [127].

Luteolin has been shown to reduce cortical Aβ deposition and ameliorate neuronal pathology in AD models. Its anti-inflammatory effects include inhibition of reactive astrogliosis and suppression of pro-inflammatory cytokines such as TNF-α, IL-1β, and IL-6, suggesting an indirect role in modulating Th17/Treg-related neuroinflammation [128,129].

Ursolic acid (UA) acts as a selective RORγt antagonist, directly inhibiting Th17 cell differentiation and IL-17 production. In addition, UA attenuates neuronal injury by correcting the MMP/TIMP imbalance associated with microglial activation. However, it should be noted that most evidence supporting UA is derived from non-AD inflammatory models, and its efficacy in AD-specific systems remains to be further validated [130].

Collectively, these compounds converge on key regulatory nodes of Th17/Treg balance, including cytokine signaling, transcriptional control, and neuroglial activation, thereby contributing to the attenuation of AD-associated neuroinflammation.

#### 5.2.3. Regulation of Oxidative Stress and Immunometabolic Balance

Oxidative stress is a key driver of Th17/Treg imbalance in AD, as excessive reactive oxygen species (ROS) production promotes pro-inflammatory signaling and disrupts immune homeostasis. Several natural compounds exert neuroprotective effects by targeting oxidative stress and associated immunometabolic pathways.

Thymol, a monoterpenoid phenol derived from Lamiaceae plants, modulates immune responses by restoring Th17/Treg balance. It promotes Treg differentiation and function while inhibiting Th17 and Th1 cell development, leading to a reduced Th17/Treg ratio. Mechanistically, thymol upregulates Foxp3 and TGF-β expression, enhances Treg immunosuppressive activity, and suppresses pro-inflammatory cytokines such as IFN-γ and IL-17, thereby reestablishing immune homeostasis [131,132].

Grape seed proanthocyanidin extract (GSPE) exerts antioxidant and anti-inflammatory effects through multiple mechanisms, including suppression of inducible nitric oxide synthase (iNOS) expression and nitric oxide (NO) production, as well as scavenging reactive species and enhancing endogenous antioxidant defenses. In addition, GSPE downregulates pro-inflammatory mediators such as TNF-α, COX-2, and p53, which are associated with Th17 activation, suggesting an indirect role in modulating Th17/Treg balance [133,134].

Epigallocatechin-3-gallate (EGCG) exhibits multi-target neuroprotective effects by modulating both amyloid metabolism and immune responses. EGCG inhibits β-site APP-cleaving enzyme 1 (BACE1) activity, thereby reducing Aβ production, and attenuates Aβ-induced neurotoxicity through regulation of glycogen synthase kinase 3 (GSK3) and inhibition of the c-Abl/FE65 signaling axis. Furthermore, EGCG reduces oxidative stress by downregulating iNOS expression and restoring intracellular antioxidant capacity, while promoting Treg activity and suppressing Th17 differentiation [135,136].

Collectively, these compounds mitigate oxidative stress and regulate immunometabolic pathways, thereby contributing to the restoration of Th17/Treg balance and attenuation of AD-associated neuroinflammation. However, most evidence is derived from non-AD or systemic inflammatory models, highlighting the need for further validation in AD-specific systems.Details of the experimental models and mechanisms of natural compounds are summarized in Table A2 and Table A3 (see in Appendix A).

### 5.3. Translational Considerations and Evidence Limitations

Notably, many of these compounds have primarily been investigated in non-AD models, including autoimmune and acute inflammatory conditions. While these studies provide valuable insights into Th17/Treg modulation, their direct applicability to AD remains uncertain.

Overall, natural compounds targeting Th17/Treg balance exhibit considerable therapeutic potential; however, their efficacy varies depending on the strength of evidence. Compounds such as ganoderic acid A, paeoniflorin, and matrine are supported by AD-specific experimental data, whereas others, including quercetin, thymol, and ursolic acid, rely largely on non-AD models. This variability highlights the need for further validation in AD-relevant systems and clinical studies.

## 6. Future Research Directions

Despite advances in Aβ- and tau-targeted therapies, effective disease-modifying treatments for AD remain limited. Current monoclonal antibodies, such as Aducanumab [137] and Lecanemab [138] for Aβ, and Semorinemab and BIIB080 [139] for tau, have demonstrated only modest clinical benefits, partly due to insufficient BBB penetration and the spatiotemporal heterogeneity of pathological targets.

These limitations highlight the need for alternative or complementary therapeutic strategies, particularly those targeting neuroinflammation and immune dysregulation. In this context, the Th17/Treg axis has emerged as a promising immunological target. Despite growing evidence supporting the therapeutic potential of natural compounds in modulating Th17/Treg balance, several critical gaps identified in this review highlight priorities for future research.

First, the limited validation of many compounds in AD-specific models represents a major barrier to translation. Future studies should prioritize evaluating these compounds in disease-relevant systems, including transgenic AD models and pathology-driven experimental platforms, to establish their efficacy in the context of AD.

Second, beyond model validation, a key challenge lies in the insufficient mechanistic integration of existing findings. While several compounds (such as ganoderic acid A, paeoniflorin, and matrine) demonstrate promising effects in AD models, their regulatory effects on Th17/Treg balance, Aβ aggregation, and tau pathology are often studied in isolation [140]. Future research should therefore focus on elucidating how these pathways interact and whether coordinated targeting of immune and pathological processes can yield synergistic therapeutic benefits.

Third, immunometabolic regulation represents an emerging but underexplored mechanism [141]. As highlighted in this review, metabolic pathways such as glycolysis, oxidative stress, and mitochondrial function play a crucial role in shaping Th17/Treg balance. Future studies should investigate whether natural compounds can coordinately modulate these immunometabolic pathways to enhance therapeutic efficacy.

Fourth, translational challenges remain a major barrier. Many natural compounds have poor bioavailability, limited BBB permeability, and rapid metabolism [142]. Addressing these pharmacokinetic limitations through structural optimization and advanced delivery systems will be essential for clinical application.

Finally, although nanotechnology-based approaches may improve drug delivery and targeting efficiency, their application in Th17/Treg modulation for AD remains largely exploratory [143]. Future research should carefully evaluate their safety, specificity, and relevance in AD models before clinical translation.

## 7. Conclusions

This review highlights the central role of Th17/Treg imbalance in AD pathogenesis, linking peripheral immune dysregulation with neuroinflammation and progressive neurodegeneration. Accumulating evidence indicates that natural compounds can modulate this axis through multiple mechanisms, including regulation of cytokine networks, signaling pathways, and immunometabolic processes.

Among the compounds discussed, ganoderic acid A, paeoniflorin, and matrine demonstrate relatively stronger evidence in AD-specific models, whereas others, such as quercetin, thymol, and ursolic acid, are primarily supported by non-AD experimental systems. This heterogeneity underscores the importance of critically evaluating the strength and relevance of existing evidence.

Despite promising preclinical findings, a major limitation is the lack of clinical validation of Th17/Treg-targeted interventions in AD patients. In addition, challenges such as poor bioavailability, limited BBB penetration, and incomplete mechanistic understanding continue to hinder translation.

Overall, targeting the Th17/Treg axis—particularly through integrated immunometabolic modulation—represents a promising but still emerging therapeutic strategy for AD. Future studies should focus on bridging the gap between preclinical research and clinical application to fully realize its therapeutic potential.

## Figures and Tables

**Figure A6:**
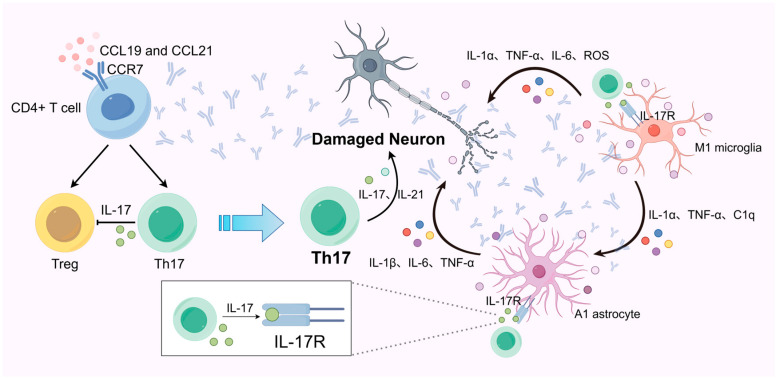
CCL21-CCR7-Mediated T Cell Brain Infiltration and IL-17 Cascade Amplify Tripartite Cellular Damage in AD.

**Figure A7:**
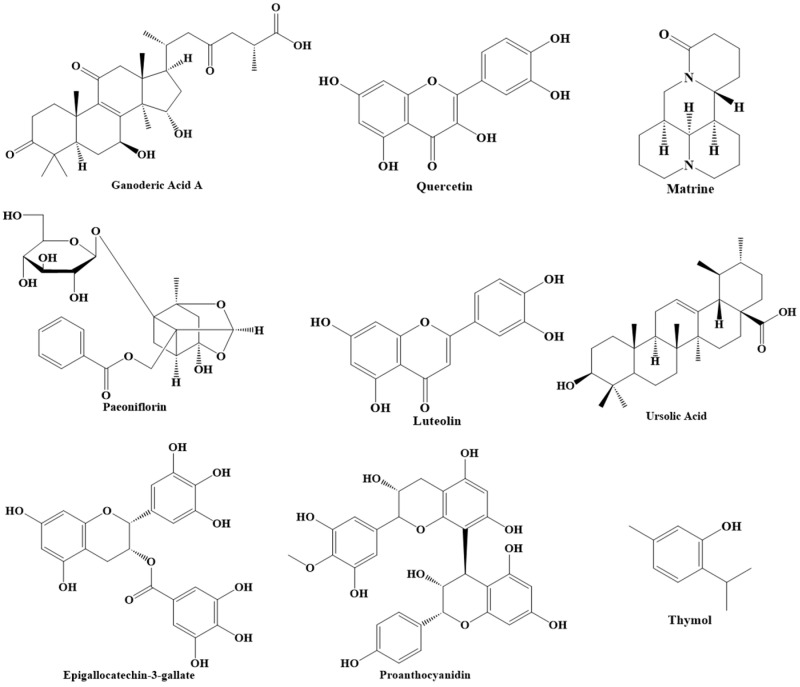
Structural formulas of natural compounds modulating Th17/Treg balance.

## Data Availability

No new data were created or analyzed in this study.

## Abbreviations

The following abbreviations are used in this manuscript:

AD	Alzheimer’s disease
ACC1	Acetyl-CoA carboxylase 1
Akt	Protein kinase B
AMPK	AMP-activated protein kinase
APCs	Antigen-presenting cells
APOE	Apolipoprotein E
AS	Astrocytes
Aβ	Amyloid-beta peptides
BACE1	β-site amyloid precursor protein cleaving enzyme 1
Bax	Bcl-2-associated X protein
BBB	Blood–brain barrier
Bcl-2	B-cell lymphoma 2
bet	T-box transcription factor TBX21
C1q	Complement component 1q
cAbl	Cellular Abelson tyrosine kinase
cAMP	Cyclic adenosine monophosphate
caspase-1	Cysteine-dependent aspartate-specific protease 1
CCL	C-C motif chemokine ligand
CCR	C-C chemokine receptor type
CD	Cluster of differentiation
Cdc42	Cell division control protein 42 homolog
CNS	Central nervous system
COX-2	Cyclooxygenase-2
DG	2-Deoxy-D-glucose
EGCG	Epigallocatechin-3-gallate
ERK	Extracellular signal-regulated kinases
FAO	Fatty acid oxidation
FoxO1	Forkhead box protein O1
Foxp3	Forkhead box protein 3
GAA	Ganoderic acid A
GSK3	Glycogen synthase kinase 3
GSPE	Grape seed proanthocyanidin extract
HIF	Hypoxia-inducible factor
ICAM-1	Intercellular adhesion molecule 1
ICER	Inducible cAMP early repressor
IDE	Insulin-degrading enzyme
IFN-γ	Interferon-γ
IL	Interleukin
iNOS	inducible nitric oxide synthase
JAK	Janus kinase
LRP1	Lipoprotein receptor-related protein 1
MAT	Matrine
MG	Microglia
MHC-II	Major histocompatibility complex class II
MMP	Matrix metalloproteinase
MMP9	Matrix metalloproteinase-9
mTOR	Mechanistic target of rapamycin
mTORC	Mechanistic target of rapamycin complex
MyD88	Myeloid differentiation primary response protein 88
NEP	Neprilysin
NFTs	Neurofibrillary tangles
NF-κB	Nuclear factor kappa-B
NGF	Nerve growth factor
NLRP3	NOD-like receptor family pyrin domain containing 3
NMDA	N-methyl-D-aspartate
OL	Oligodendrocytes
OXPHOS	Oxidative phosphorylation
p53	Tumor protein p53
PF	Paeoniflorin
PI3K	Phosphoinositide 3-kinase
p-JAK2	Phosphorylated Janus kinase 2
p-STAT3	Phosphorylated signal transducer and activator of transcription 3
RORγt	Retinoic acid-related orphan receptor gamma t
ROS	Reactive oxygen species
STAT	Signal transducer and activator of transcription
TGF-β	Transforming growth factor-β
Th17	T helper 17 cells
TIMP	Tissue inhibitor of metalloproteinases
TLRs	Toll-like receptors
TNF-α	Tumor necrosis factor-α
Treg	regulatory T cells
TREM2	Triggering receptor expressed on myeloid cells 2
TYK2	Tyrosine kinase 2
UA	Ursolic acid
VCAM-1	Vascular cell adhesion molecule-1

## Appendix A

### Appendix A.1

**Table A1:** The role of cytokines in the balance of Th17/Treg.

Cytokines	Mechanism	Change
TGF-β	Induces Smad2/3 in naive CD4^+^ T cells to activate Foxp3 transcription.	Treg ↑ [45] Th17 ↑ [46]
	Activates Smad3/4 to drive RORγt expression in Th17.	
IL-2	IL-6R subunit downregulation inhibits Th17 differentiation, while STAT5-dependent GM-CSF upregulation drives Th17 to pTh17.	pTh17 ↑ [47,144] Treg ↑ [53]
	Low-dose IL-2 modulates immunity via Treg differentiation.	
IL-6	STAT3 phosphorylation drives Th17 gene signature (RORγt/IL-17/IL-23).	Th17 ↑ [49]
IL-15	Reduces IL-17 secretion to inhibit Th17 differentiation.	
IL-18	MyD88-dependent IL-1R signaling blockade suppresses Th17 differentiation	Th17 ↓ [54]
IL-21	STAT3 activation drives Th17 differentiation through RORγt upregulation.	Th17 ↓ [52]
IL-23	Th17 transcriptional signature upregulation sustains differentiation commitment.	Th17 ↑ [145]
HIF-1α	RORγt transcriptional activation promotes Th17 differentiation, whereas targeted Foxp3 degradation inhibits Treg commitment.	Th17 ↑ Treg ↓ [49]

↑ indicates increase; ↓ indicates decrease.

### Appendix A.2

**Table A2:** Experimental models of natural compounds targeting Th17/Treg-mediated neuroinflammation in AD.

Chemical Compound	Model Type	In Vivo/Vitro Model
Ganoderic Acid A	AD model	D-galactose-induced AD mice
Quercetin	Non-AD model	SJL/J mice
		SJL/J mouse splenocyte
Paeoniflorin	Non-AD model	BALB/c and C57BL/6 mice
		Naive CD4^+^ T cells isolated from C57BL/6 mouse spleens.
Matrine	AD model	SD rats with hippocampal Aβ1-42 injection
Luteolin	AD model	3 × Tg-AD mice and wild-type (WT, C57BL/6J) mice
Ursolic Acid	Non-AD model	EAE mice
		human and mouse CD4^+^ T cells
Thymol	Non-AD model	Splenocytes from 6- to 8-week-old healthy female BALB/c mice
GSPE	Non-AD model	Male DBA/1J mice
		DBA/1J mouse splenocyte

**Table A3:** Mechanisms and Th17/Treg-related markers of natural compounds targeting neuroinflammation in AD.

Chemical Compound	Mechanism	Th17/Treg Markers
Ganoderic Acid A	JAK2/STAT3 inhibition suppresses pro-inflammatory mediators while enhancing immunoregulatory factors	Treg ↑, Th17 ↓ [120]
Quercetin	Blocks IL-12-induced JAK2/TYK2 phosphorylation to suppress STAT3/4 activation	Th1 ↓, IL-6 ↓ IL-17 ↓ [124,126]
Paeoniflorin	Suppresses Th17 cytokine transcription and inhibits STAT3 phosphorylation in Th17 polarization	Th17 ↓, IL-17 ↓ [121,123]
Matrine	Downregulates RORγt/IL-17A/IL-23 axis while upregulating Foxp3-driven immunoregulation	Treg ↑, Th17 ↓ [127]
Luteolin	Suppresses ER stress proteins and astrocyte hyperactivation, while inhibiting pro-inflammatory cytokines	TNF-α, IL-6 ↓ [128]
Ursolic Acid	Targeted suppression of RORγt and IL-17 in Th17-mediated immunity	Th17 ↓ [130]
Thymol	Modulating of Immunoregulatory vs Pro-inflammatory Transcriptional Network	Treg ↑, Th17 ↓ [131,132]
GSPE	STAT3-dependent Th17 cytokine suppression with concurrent Treg induction	Treg ↑, Th17 ↓ [134]

↑ indicates increase; ↓ indicates decrease.
